# A phase II study of the safety of olanzapine for oxaliplatin based chemotherapy in coloraectal patients

**DOI:** 10.1038/s41598-021-84225-6

**Published:** 2021-02-25

**Authors:** Junichi Nishimura, Akiko Hasegawa, Toshihiro Kudo, Tomoyuki Otsuka, Masayoshi Yasui, Chu Matsuda, Naotsugu Haraguchi, Hajime Ushigome, Nozomu Nakai, Tomoki Abe, Hisashi Hara, Naoki Shinno, Kei Asukai, Shinichiro Hasegawa, Daisaku Yamada, Keijiro Sugimura, Kazuyoshi Yamamoto, Hiroshi Wada, Hidenori Takahashi, Takeshi Omori, Hiroshi Miyata, Masayuki Ohue

**Affiliations:** 1 Department of Gastroenterological Surgery, Osaka International Cancer Institute, 3-1-69 Otemae, Chuo-ku, Osaka, Osaka 541-8567 Japan; 2 Department of Clinical Oncology, Osaka International Cancer Institute, 3-1-69 Otemae, Chuo-ku, Osaka, 541-8567 Japan

**Keywords:** Colorectal cancer, Chemotherapy

## Abstract

Olanzapine has exhibited efficacy as an antiemetic agent when used with 5-HT_3_ receptor antagonists, dexamethasone, and NK_1_ receptor antagonists for patients receiving highly emetogenic chemotherapy. In addition, several studies have reported the efficacy or safety of olanzapine in patients receiving moderately emetogenic chemotherapy, including carboplatin, irinotecan, and oxaliplatin. However, no reports of olanzapine use have focused on patients receiving oxaliplatin-based chemotherapy. Therefore, we analyzed the safety of antiemetic therapy using olanzapine, palonosetron, aprepitant, and dexamethasone in colorectal cancer patients undergoing oxaliplatin-based chemotherapy. This study was a prospective phase II single-institution study of 40 patients (median age 60 years, 23 patients were male). The primary endpoint was the incidence of adverse events, and the exploratory endpoints were the rate of chemotherapy-induced nausea and vomiting. Almost all patients (90%) had a performance status of 0. All patients received the scheduled antiemetic therapy. The most common adverse event was somnolence (n = 7 patients, 17.5%). All adverse events were grade 1. Thirty-six patients were included in the exploratory analysis of efficacy. No patients experienced vomiting during the first 120 h after chemotherapy, and complete response and complete control were both 86.1%. The rate of total control was 55.6% during the same time period. Olanzapine use with 5-HT_3_ receptor antagonists, dexamethasone, and NK_1_ receptor antagonists was safe for colorectal cancer patients receiving oxaliplatin-based chemotherapy.

## Introduction

Chemotherapy-induced nausea and vomiting (CINV) are common adverse events of cancer chemotherapy. Previously, we reported a non-blinded, randomized, controlled phase 3 study (SENRI trial) that explored the efficacy of an antiemetic regimen of 5-hydroxytryptamine type 3 (5-HT_3_) receptor antagonists, dexamethasone, and neurokinin-1 (NK_1_) receptor antagonists for colorectal cancer patients receiving oxaliplatin-based chemotherapy^1,2^. The rate of vomiting was significantly reduced in the aprepitant group that received 5-HT_3_ receptor antagonists, dexamethasone, and NK_1_ receptor antagonists. We also reported a subgroup analysis for the SENRI trial; we found gender to be a risk factor for vomiting and nausea. However, in the SENRI trial, the rate of no nausea and no vomiting without rescue medication was 58.3%, which is still low, in the first 120 h after chemotherapy. To reduce CINV during oxaliplatin-based chemotherapy, new agents can be added to the 5-HT_3_ receptor antagonists, dexamethasone, and NK_1_ receptor antagonists.

Olanzapine is an antipsychotic agent that blocks multiple neurotransmitters, including dopamine at D1, D2, D3, and D4 receptors; serotonin at 5-HT type 2a, 5-HT type 2c, 5-HT_3_, and 5-HT type 6 receptors; catecholamines at alpha1-adrenergic receptors; acetylcholine at muscarinic receptors; and histamine at H1 receptors in the central nervous system. Since Passik et al. first reported the pilot study of olanzapine as antiemetic therapy^3^, work has focused on olanzapine activity as an antiemetic agent.

Several studies have reported that regimens including olanzapine are better than triplet-combination regimens (5-HT_3_ receptor antagonists, dexamethasone, and NK_1_ receptor antagonists)^4–6^. The guidelines established by the American Society of Clinical Oncology (ASCO) and National Comprehensive Cancer Network (NCCN) recommend olanzapine with the triplet-combination therapy against highly emetogenic chemotherapy (HEC) regimens^7,8^.

For moderately emetogenic chemotherapy (MEC), a small randomized control trial has reported the effects of olanzapine as an antiemetic agent in addition to 5-HT_3_ receptor antagonists, dexamethasone, and NK_1_ receptor antagonists. They could not confirm the efficacy of olanzapine at the acute phase of emesis because of a small sample size and did not focus on a regimen including oxaliplatin^5^. In this phase 2 study, we assessed the safety of antiemetic treatment using olanzapine, 5-HT_3_ receptor antagonists, dexamethasone, and NK_1_ receptor antagonists. We also explored the rate of CINV in patients receiving oxaliplatin-based chemotherapy.

## Patients and methods

### Eligibility criteria

This study is registered at UMIN Clinical Trials Registry (number UMIN 000032591). The study included oxaliplatin-naïve patients with colorectal cancer who were 20 years of age or older and received FOLFOX (oxaliplatin 85 mg/m^2^, levo-leucovorin 200 mg/m^2^, and fluorouracil 400 mg/m^2^ bolus and 2400 mg/m^2^ continuous infusion) or XELOX (capecitabine 2000 mg/m^2^ orally on days 1–14 and oxaliplatin 130 mg/m^2^ via intravenous infusion on day 1). Molecular targeted therapy was allowed. Neoadjuvant chemotherapy, recurrent/unresectable chemotherapy, and adjuvant chemotherapy were included in this study. Institutional review boards approved the study protocol, and written informed consent was obtained from all participants before enrollment.

### Exclusion criteria

Patients were excluded if they had nausea or vomiting within 24 h prior to enrollment, were treated with antiemetics within 24 h prior to treatment, had any factors causing nausea or vomiting (e.g., gastrointestinal obstruction or symptomatic brain metastasis), had uncontrollable diabetes, were pregnant or lactating or planning on becoming pregnant, or were currently receiving pimozide.

### Treatment protocol

All patients received oral aprepitant (125 mg) plus an intravenous 5-HT_3_ receptor antagonist (palonosetron hydrochloride) and dexamethasone (6.6 mg) on day 1, and aprepitant (80 mg) and oral dexamethasone (2 mg) twice daily on days 2 and 3. Olanzapine (5 mg) was administered after dinner for 4 days from the first day of oxaliplatin administration.

### Patient evaluation

The study period was only the first cycle of oxaliplatin-based chemotherapy. Patients recorded their response in a diary daily for 5 days. The use of rescue therapy, defined as any medication taken to treat nausea and vomiting, was also recorded. The severity of nausea was classified into four grades: 0, none; 1, mild; 2, moderate; or 3, severe. No nausea and mild nausea were considered as no significant nausea. For vomiting, patients recorded the frequency. Retches were also included as vomiting^1^. An emetic episode was defined as single or multiple emetic vomiting experiences occurring within an interval of 5 min. It was also subjectively assessed by each patient.

### Statistical analysis

The primary endpoint was the incidence of grade 3 or 4 adverse events. Thirty-two patients were required to achieve 80% statistical power with a one-sided α error of 0.05 assuming an expected adverse event rate of 1% and a threshold of 10%. An arc-sine transformation was performed with the binomial test for the required sample sizes calculation^9^. The planned sample size was 40 patients for enrollment after taking dropouts into consideration. The exploratory endpoint was the rate of no vomiting, no nausea, no significant nausea (defined as none or mild nausea), complete response (defined as no vomiting episode and no rescue therapy), complete control (defined as no vomiting, no rescue therapy, and no significant nausea), and total control (no vomiting, no rescue therapy, and no nausea) in the acute (first 24 h after chemotherapy), delayed (24–120 h after chemotherapy), and overall phases (0–120 h after chemotherapy). We calculated 95% confidence intervals (CIs) for the proportion of patient characteristics and CINV. Adverse events were graded using Common Toxicity Criteria for Adverse Events, version 4.0. Alcohol use was divided into two groups: drinker, patient has drinking habit every day, and light/non-drinker for others. Statistical analyses were performed using JMP software version 13 (SAS Institute, Cary, NC, USA). The estimation of sample size was carried out in R version 4.0.2.

### Ethical approval

All procedures involving human participants were performed in accordance with the ethical standards of the institutional and/or national research committee and with the 1964 Helsinki declaration and its later amendments or comparable ethical standards. This study was approved by the Institutional Review Board of Osaka International Cancer Center (approval no. 18015) and was registered at UMIN Clinical Trials Registry (number UMIN000032591) on 15/05/2018.

## Results

Forty patients (age range 36–80 years; 23 males) were enrolled in this study between March 2018 and April 2019. Patient characteristics are provided in Table 1. The median age was 60 years ranged 36 to 80 years. The male was 23 (58%). Most patients had a performance status of 0. Twelve patients (30%) received FOLFOX regimen, and 28 patients (70%) received the CAPOX regimen. The use of molecular target drug was in 13 patients (33%). The previous colon resecton surgery was done in 30 patients (83%). Ileostomy was underwent in 4 patients (11%), and colostomy in 3 patients (8%). Only 1 patient (3%) received chemotherapy previously. The reason of chemotherapy was preoperative neoadjuvant chemotherapy in 2 patients (5%), postoperative adjuvant chemotherapy in 19 patients (48%), and chemotherapy for unresectable or recurrence tumor in 19 patients (48%). Alcohol use was light or nondrinker in 30 patients (75%) and drinker in 10 patients (25%). History of vomiting during pregnancy was noted in 8 of 16 patients (50%). History of motion sickness was observed in 5 patients (13%).

**Table 1:** Patient characteristics.

Characteristic	All patients (n = 40)
Median age, years (IQR)	60 (53–66)
Sex, male	23 (58, 42–71)
**Ethnicity, n (%)**	
Asian	40 (100)
**Performance status**	
0	36 (90, 77–96)
1	3 (8, 3–20)
2	1 (3, 0.4–13)
**Chemotherapy regimen**	
FOLFOX	12 (30, 18–45)
CAPOX	28 (70, 55–82)
**Use of molecular target drug**	
Yes	13 (33, 20–48)
**Colorectal resection**	
Yes	34 (85, 71–93)
**Stoma**	
Ileostomy	5 (13, 5–26)
Colostomy	3 (8, 3–20)
**Purpose of chemotherapy**	
Neoadjuvant treatment	2 (5, 1–17)
Adjuvant treatment	19 (48, 33–63)
Metastatic disease	19 (48, 33–63)
**Smoking history**	
Never	19 (48, 33–63)
Ex-smoker	21 (52, 37–67)
**Alcohol use**	
Light/non-drinker	30 (75, 60–86)
Drinker	10 (25, 14–40)
History of vomiting during pregnancy	8 (47, 26–69)
History of motion sickness	5 (13, 5–26)

*IQR* interquartile range.

Data are given as n (%, confidence interval) unless otherwise noted.

### Adverse events

All patients received the scheduled antiemetic treatment. The adverse events are given in Table 2. The most common adverse events were anorexia (35%) and somnolence (17.5%). All adverse events were grade 1 and were tolerated. We did not observe any adverse hematological events, and no treatment-related deaths were reported during this study.

**Table 2:** Adverse events in the study population.

Event	n (%, CI)
Anorexia	14 (35, 22–50)
Somnolence	7 (17.5, 9–32)
Constipation	1 (2.5, 0.4–13)
Diarrhea	1 (2.5, 0.4–13)
Malaise	1 (2.5, 0.4–13)
Hematological toxicity	0 (0.0, 0–9)
Hepatotoxicity	0 (0.0, 0–9)
Nephrotoxicity	0 (0.0, 0–9)

*CI* 95% confidence interval.

All adverse events were Grade 1.

### Efficacy

Thirty-six patients recorded their responses in the diary. Four patients forgot to record the patient diary. Thus, 36 of the 40 patients were included in the analysis of efficacy (Table 3). None of the patients experienced vomiting in the acute, delayed, or overall phases (Table 4). Five patients (14%) used rescue antiemetic therapy. Only 2 patients (6%) who had significant nausea used rescue antiemetic therapy. The proportions of patients with each outcome in each phase are given in Table 3. No nausea in the acute, delayed, and overall phase was noted in 97.2%, 61.1%, and 61.1% of patients, respectively.

**Table 3:** Characteristics of patients included in the CINV analysis.

Characteristic	All patients (n = 36)
Median age, years (IQR)	59 (52–65)
Sex, male	20 (56, 40–70)
**Performance status**	
0	33 (92, 78–97)
1	2 (6, 2–18)
2	1 (3, 0.5–14)
**Chemotherapy regimen**	
FOLFOX	10 (28, 16–44)
CAPOX	26 (72, 56–84)
**Use of molecular target drug**	
Yes	12 (33, 20–50)
**Colorectal resection**	
Yes	30 (83, 68–92)
**Stoma**	
Ileostomy	4 (11, 4–25)
Colostomy	3 (8, 3–22)
**Purpose of chemotherapy**	
Neoadjuvant treatment	2 (6, 2–18)
Adjuvant treatment	18 (50, 34–66)
Metastatic disease	16 (44, 30–60)
**Smoking history**	
Never	17 (47, 32–63)
Ex-smoker	19 (53, 37–68)
**Alcohol use**	
Light/non-drinker	28 (78, 62–88)
Drinker	8 (22, 12–38)
History of vomiting during pregnancy	8 (50, 28–72)
History of motion sickness	5 (14, 6–29)

*IQR* interquartile range.

Data are given as n (%, confidence interval) unless otherwise noted.

**Table 4:** Efficacy analysis in 36 patients.

Phase	Acute % (CI)	Delayed % (CI)	Overall % (CI)
No vomiting	100 (90–100)	100 (90–100)	100 (90–100)
No nausea	97.2 (86–100)	61.1 (45–75)	61.1 (45–75)
No significant nausea	100 (90–100)	94.4 (82–98)	94.4 (82–98)
Complete response	100 (90–100)	86.1 (71–94)	86.1 (71–94)
Complete control	100 (90–100)	86.1 (71–94)	86.1 (71–94)
Total control	97.2 (86–100)	55.6 (40–70)	55.6 (40–70)

*CI* 95% confidence interval.

## Discussion

In this study, we analyzed the safety of olanzapine as an antiemetic agent with 5-HT_3_ receptor antagonists, dexamethasone, and NK_1_ receptor antagonists for colorectal cancer patients undergoing oxaliplatin-based chemotherapy. The incidence of somnolence was 17.5%, and the patients suffered no grade 2 or greater adverse events. We also analyzed the rate of CINV. None of the patients experienced vomiting, and a complete response occurred in 86.1% of patients in the delayed and overall phases. To the best of our knowledge, this report is the first to analyze the safety and efficacy of adding olanzapine to triple antiemetic therapy in oxaliplatin-based chemotherapy.

In HEC regimens, several studies have shown the efficacy of olanzapine. Navari et al. first reported the efficacy of adding olanzapine to dexamethasone and granisetron^4^. They also reported the efficacy of olanzapine combined with an NK_1_ receptor antagonist, a 5-HT_3_ receptor antagonist, and dexamethasone^10^. The FOND-O trial reported the efficacy of adding olanzapine to fosaprepitant, ondansetron, and dexamethasone in patients undergoing HEC and hematopoietic transplantation^11^. Recently, Hashimoto et al. assessed adding olanzapine to aprepitant, palonosetron, and dexamethasone in patients receiving cisplatin-based chemotherapy^6^.

For MEC regimens, only one small randomized trial has evaluated the efficacy of olanzapine with palonosetron and dexamethasone, without NK_1_ receptor antagonist. Their study did not meet the primary endpoint of complete response, but the rate of significant nausea was significantly reduced in the olanzapine-treated group. Tanaka et al. reported a phase II study of the addition of olanzapine to antiemetic therapy with aprepitant, a 5-HT3 receptor antagonist, and dexamethasone in patients receiving chemotherapy including carboplatin^12^. In their study, the complete response rate in the overall phase was 93.9%, compared to 86.1% in the present study. In the SENRI trial, which was a non-blinded randomized trial, the complete response rate of patients treated with NK_1_ receptor antagonist, a 5-HT_3_ receptor antagonist, and dexamethasone was 85.0%. It was not possible to unconditionally compare the SENRI trial to this study; the number was small, evaluation was difficult, and we could not confirm the effectiveness of adding olanzapine determined by the complete response. Adding olanzapine may increase the complete response rate, which must be proven in a future phase III trial.

Several studies have investigated the efficacy and safety of 5 mg olanzapine with an NK_1_ receptor antagonist, 5-HT_3_ receptor antagonist, and dexamethasone in Japanese patients undergoing HEC^13–15^. A double-blind randomized phase II study suggested a recommended dose of 5 mg olanzapine as an antiemetic agent^15^. Therefore, we administered 5 mg olanzapine, not 10 mg, in this study. We did not note any severe adverse events, including somnolence. Thus, administration of 5 mg olanzapine to NK_1_ receptor antagonist, palonosetron, and dexamethasone is a safe alternative to antiemetics for colorectal cancer patients treated with oxaliplatin-based chemotherapy.

In this study, we administered palonosetron, a second generation 5-HT_3_ receptor antagonist characterized by a long plasma elimination half-life (~ 40 h) and a high selective binding affinity for the 5-HT_3_ receptor. Several studies have shown a superior antiemetic effect of palonosetron compared to other 5-HT_3_ receptor antagonists^16–19^. The efficacy of palonosetron relative to other 5-HT_3_ receptor antagonists has been evaluated in a meta-analysis^20^, and the efficacy of the combination of NK_1_ receptor antagonist, palonosetron, and dexamethasone compared to other antiemetic regimens was evaluated recently in a meta-analysis^21^. The combination antiemetic therapy of NK_1_ receptor antagonist, palonosetron, and dexamethasone was superior in the delayed phase with MEC regimen, and the overall phase for HEC and MEC regimens. The TRIPLE study, a randomized, double-blind, phase III trial, revealed that the complete response in the delayed period was higher in patients using palonosetron than patients using granisetron^22^.

The present study has some limitations. First, it was a single-arm trial with limited sample size at a single institution. Second, we assessed the safety of olanzapine in patients receiving oxaliplatin-based chemotherapy, but the efficacy of this antiemetic regimen was unclear. Further comparative studies are needed to determine the efficacy.

In conclusion, administration of olanzapine with NK1 receptor antagonist, palonosetron, and dexamethasone is a safe alternative to antiemetics for colorectal cancer patients treated with oxaliplatin-based chemotherapy.
